# Advantage of ostarine over raloxifene and their combined treatments for muscle of estrogen-deficient rats

**DOI:** 10.1007/s40618-023-02188-z

**Published:** 2023-09-06

**Authors:** P. J. Roch, L. Noisser, K. O. Böker, D. B. Hoffmann, A. F. Schilling, S. Sehmisch, M. Komrakova

**Affiliations:** 1 Department of Trauma Surgery, Orthopaedics and Plastic Surgery, University of Göttingen, Robert-Koch-Str. 40, 37075 Göttingen, Germany; 2 Department of Trauma Surgery, Hannover Medical School, University of Hannover, Carl-Neuberg-Str. 1, 30625 Hannover, Germany

**Keywords:** Ovariectomized rat model, Selective androgen receptor modulators (SARMs), Selective estrogen receptor modulators (SERMs), Muscle, Bone

## Abstract

**Purpose:**

Selective androgen (ostarine, OST) and estrogen (raloxifene, RAL) receptor modulators with improved tissue selectivity have been developed as alternatives to hormone replacement therapy. We investigated the combined effects of OST and RAL on muscle tissue in an estrogen-deficient rat model of postmenopausal conditions.

**Methods:**

Three-month-old Sprague Dawley rats were divided into groups: (1) untreated non-ovariectomized rats (Non-OVX), (2) untreated ovariectomized rats (OVX), (3) OVX rats treated with OST, (4) OVX rats treated with RAL, (5) OVX rats treated with OST and RAL. Both compounds were administered in the diet. The average dose received was 0.6 ± 0.1 mg for OST and 11.1 ± 1.2 mg for RAL per kg body weight/day. After thirteen weeks, rat activity, muscle weight, structure, gene expression, and serum markers were analyzed.

**Results:**

OST increased muscle weight, capillary ratio, insulin-like growth factor 1 (Igf-1) expression, serum phosphorus, uterine weight. RAL decreased muscle weight, capillary ratio, food intake, serum calcium and increased Igf-1 and Myostatin expression, serum follicle stimulating hormone (FSH). OST + RAL increased muscle nucleus ratio, uterine weight, serum phosphorus, FSH and luteinizing hormone and decreased body and muscle weight, serum calcium. Neither treatment changed muscle fiber size. OVX increased body and muscle weight, decreased uterine weight, serum calcium and magnesium.

**Conclusion:**

OST had beneficial effects on muscle in OVX rats. Side effects of OST on the uterus and serum electrolytes should be considered before using it for therapeutic purposes. RAL and RAL + OST had less effect on muscle and showed endocrinological side effects on pituitary–gonadal axis.

## Introduction

The aging of the world's population poses serious medical, social, and economic challenges. In postmenopausal women, hormonal changes such as estrogen decline contribute to the development of sarcopenia and osteoporosis [1, 2]. Sarcopenia describes the coexistence of reduced muscle quality or quantity and physical limitations [3].

Direct hormone replacement therapy is essential in certain cases of hormone deficiency in men and women [4–6]. Nevertheless, severe side effects such as thromboembolism have prompted the development of selective androgen and estrogen receptor modulators (SARMs and SERMs, respectively) with higher tissue selectivity [5, 7, 8].

The biochemical hypothesis for the superior bioavailability and pharmacokinetic profile of SARMs compared to testosterone is the resisted aromatization of 5-α-reduction [9]. However, while SARMs are not yet approved [10], SERMs have been shown to be a safe therapeutic option for postmenopausal symptoms with fewer side effects compared to estrogen [7].

The SARM ostarine (OST), also known as S-22, MK-2866, enobosarm or GTx-024, showed increased vascularization and citrate synthase activity in skeletal muscle in a rat model of postmenopausal osteoporosis [11], and beneficial effects on muscle in orchiectomized rats [12]. In addition, clinical studies have shown improved physical function and beneficial effects on body mass and muscle in elderly men and postmenopausal women, and reduced muscle wasting in cancer patients [13–15].

Similarly, the SERM raloxifen (RAL) was found to increase lean body mass in postmenopausal women [16] and improve body composition in orchiectomized rats [12]. In mice of both sexes suffering from muscular dystrophy, skeletal muscle function and structure were improved by RAL [17]. More recently, the combination of OST and RAL showed equivalent effects on muscle in terms of weight gain in the levator ani muscle compared to OST alone in an orchiectomized rat model, but reduced the androgenic potential of OST in the prostate [12].

Studies on the effects of combined OST and RAL treatment on muscle structure and metabolism in the female organism are lacking. Therefore, the present study was conducted to investigate the effects of the combination of OST and RAL on skeletal muscle and metabolism in an established rat model of postmenopausal conditions and to compare it with OST and RAL treatments alone. The treatments were used as a phrophylaxis against the detrimental changes under hormone deficiency. Potential side effects were analyzed.

## Materials and methods

### General procedures

**Fig. 1:**
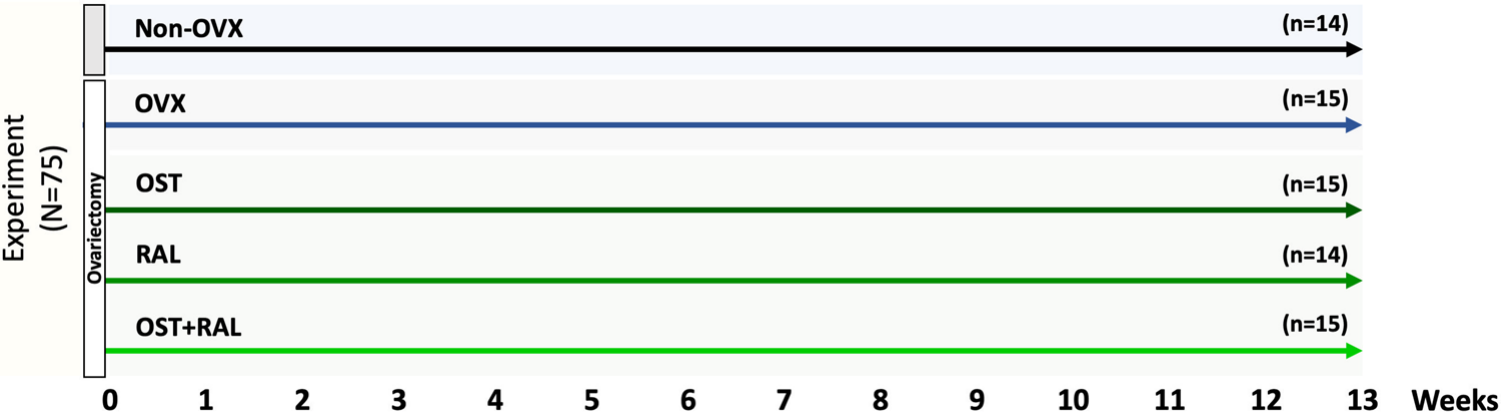
Schematic flowchart of the experiment. Eight-month-old male rats were either ovariectomized (OVX) or left intact (Non-OVX). Immediately after surgery, OVX rats were either left untreated or treated with OST, RAL or OST + RAL. (*N*) Number of rats at the beginning of the experiment, (*n*) the number of rats analyzed at the end of the experiment

The animal study protocol was approved by the local regional government (14/1396, Oldenburg, Germany) prior to the study. Seventy-five three-month-old Sprague Dawley rats (Fa. Janvier Labs, Saint-Berthevin, France) were used in the experiment. The experiment was conducted as depicted in Fig. 1. All rats were anesthetized with isoflurane, microchips (1,25 × 7 mm, ISO11784/11785, Med Associates, Inc. Fairfax, Virginia, USA) were injected s.c. for further identification of the rats, and the rats were either bilaterally ovariectomized (OVX) or left non-ovariectomized to serve as intact controls (Non-OVX). Thereafter, rats were divided into five groups, each of 15 rats: Group 1, Non-OVX; Group 2, OVX, and Groups 3 to 5, OVX rats treated with OST, RAL, or a combination of both (RAL + OST), with a dosage of 0.4 mg/kg BW for OST and 7 mg/kg BW for RAL for up to 13 weeks. The dosages were taken from the previous studies [11, 18]. Three to four rats were housed in one cage (Type Makrolon® IV, Techniplast Deutschland GmbH, Hohenpreißenberg, Germany). The rats had free access to demineralized water and soy-free pelleted food (ssniff Spezial Diät GmbH, Soest, Germany). In the latter, OST and RAL were supplied. OST was obtained from Shanghai Biochempartner Co., Ltd. (Shanghai, China), and RAL was obtained from Eli Lilly and Company (Evista®, Indianapolis, USA). Food intake and BW were weekly recorded. The average daily food intake of a rat was calculated by dividing the food consumed by the number of rats in a cage and it served for the calculation of the drug intake (OST, RAL, OST + RAL) [19]. The average received dose was 0.6 ± 0.1 mg/kg/day BW for OST and 11.1 ± 1.2 mg/kg/day for RAL.

At the end of the experiment, 13 weeks after OVX, an open field activity test was performed. Four animals at a time were placed in a cage with the bottom divided into 6 squares using a black marker and filmed with a camera (Nikon D5600, Tokyo, Japan) for 5 min. The number of completely crossed lines (horizontal movement activity, transitions), the number of uprights (vertical movement activity, rearing), and the cleaning activity (grooming) were then counted on a computer [20]. Blood samples were then collected by heart puncture under deep isoflurane anesthesia and stored at − 20 °C for further analysis. The uterus and three muscles were removed bilaterally: gastrocnemius (GM), longissimus (LM), and soleus (SM). GM and SM were weighed. All muscles were snap frozen in liquid nitrogen and stored at − 80 °C for either histological or mRNA expression analysis.

### Histological analysis

A cryotome was used to cut cross-sections of 12-µm thickness serially from the middle part of each frozen muscle (CM 1900; Leica Microsystems, Wetzler, Germany). Until staining, specimens were air dried and stored at − 20 °C. Unless otherwise indicated, all chemicals were obtained from Merck KGaA (Darmstadt, Germany).

Staining of muscle capillaries was performed using a periodic acid–Schiff (PAS) method [21]. Briefly, section was fixed in ethanol/chloroform/glacial acid solution (16:3:1), then incubated in 0.3% α-amylase from porcine pancreas, (Sigma–Aldrich Laborchemikalien GmbH, Seelze, Germany), stained using Schiff’s reagent solution (Roth, Karlsruhe, Germany) and finally treated with a 10% potassium sulfite solution. To avoid overstaining, Schiff’s reagent solution was applied under visual control (2–25 min).

For staining of muscle fibers, a modified staining method with adenosine-triphophatase (ATPase) was applied as described by Horák [22]: Sections were fixed in a solution of 1% paraformaldehyde solution (pH 6.6), 1% CaCl_2_ and 6% sucrose and then stained by an incubation in a reduced nicotinamide adenine dinucleotide diaphorase solution (pH 7.4). At last, an acidic incubation (pH 4.2) and incubation in adenosine-5´-triphosphate solution (pH 9.4) were performed [22].

Using hematoxylin eosin (HE) staining, nuclei were analyzed [23]. After muscle sections have been fixed with acetone, Mayer’s hematoxylin solution was used to stain the nuclei, and eosin G solution to stain the muscle fibers.

Aquatex® (Merck) was used to mount sections for fiber type and capillary stainings, whereas Eukitt® (Kindler GmbH, Freiburg, Germany) was taken for sections with nucleus staining. Muscle sections were analzed using a microscope (Eclipse E 600 microscope; Nikon, Tokyo, Japan), a digital camera (DS-Fi2 Digital Camera; Nikon Instruments Europe, Amsterdam, Netherlands) and a software (NIS-Elements AR 4.0 imaging software; Nikon Instruments Europe) at tenfold magnification. For the evaluation of muscle fibers, we used three randomly chosen fields of 1 mm^2^ within each ATPase-stained section. In these fields, 90 slow-twitch oxidative and fast-twitch oxidative (fiber types I and IIa, respectively) STO + FTO and 90 fast-twitch glycolytic (FTG; fiber type IIb) fibers were skirted [24]. In the ML, the fiber distribution was determined, as in this muscle, fibers show a homogenous distribution pattern [25]. In contrast, the GM showed a relative heterogenic distribution of fiber types, whereas the SM mainly consists mostly of STO fibers [26, 27]. Hence, in the latter only STO fibers were measured. The percentage of STO + FTO and FTG fibers was determined within 1 mm^2^ field. The ratio of capillaries to fibers (capillary density) as well as the ratio of nuclei to fibers (nucleus density) were calculated in two randomly chosen fields of 0.5 mm^2^ each withih the cross section [28].

### Serum analysis

For analyses of enzyme activities and electrolyte concentrations an automated chemistry analyzer Architect c16000 (Abbott, Wiesbaden, Germany) and commercially available kits (Abbott) were used at the Department of Clinical Chemistry, University of Goettingen according to the manufacturer’s instructions (Abbott). Activity of creatine kinase (CK), and concentration of calcium (Ca), magnesium (Mg), and phosphorus (P) in serum were determined. The following methods were applied: Ca and Mg: quantification by arsenazi III dye was used (7D61-20 and 7D70-30, Abbott); P: ammonium molybdate method (7D71-30, Abbott); CK: reactivator method with *N*-acetyl-*L*-cysteine (7D63-30, Abbott) [11]. Enzyme Immunoassay kit for rats (Cloud-Clone Corp., Katy, Texas, USA) was used to determine follicle stimulating hormone (FSH) and luteinizing hormone (LH) levels.

### Gene expression analysis

GM samples (100 mg; *n* = 5/group) were homogenized in 750 µl TRIzol (Thermo Fischer Scientific, WA, USA) using 4 mm tungsten carbide beads (Cat. No. 69997 Qiagen, Germany) with the aid of the Tissuelyzer LT system (Qiagen, Germany). Thereafter, the samples were incubated for 5 min at room temperature and further RNA extraction was processed according to the manufacturer’s protocol (Trizol, Thermo Fischer Scientific) using chloroform and isopropanol treatments and ethanol washings. Finally, the RNA pellet was dissolved in 20 µL H_2_O, measured by DeNovix DS-11 FX + System (DeNovix, NC, USA) and stored at − 80 °C for further analysis.

Reverse transcription was performed with 1000 ng of total RNA using an iScript cDNA synthesis kit (Biorad, CA, USA). Quantitative real-time Polymerase chain reaction (PCR) was performed on the CFX96 Real-time PCR Detection System (Biorad, CA, USA) using a SYBR Green (Biorad, CA, USA) detection marker. Relative expressions of beta-2-microglobulin (B2M), androgen receptor (Ar), estrogen receptor alpha (Er alpha), Myostatin, insulin-like growth factor 1 (Igf-1), and vascular endothelial growth factor B (Vegf-B, [29]) were measured in triplicate and effects were calculated using the 2^−ΔΔCT^ method [30]. Ready-to-use primers for B2M, Ar, Er-alpha, Igf-1 and Myostatin were obtained from Qiagen (QuantiTect Primer Assays, Qiagen, Hilden, Germany). Primers for Vegf-B were used with the following sequences: Forward GCCAGACAGGGTTGCCATAC, Reverse GGAGTGGGATGGATGATGTCAG. B2M was taken as a reference gene. We failed to measure the mRNA expression of Er beta, confirming its low and nearly undetectable expression in rodent muscle [31].

### Statistical analysis

Statistical analyses were performed using GraphPad Prism ver. 8.2.1 (GraphPad Software, San Diego, CA, USA). One-way analysis of variance (ANOVA) was applied. Differences between groups were analyzed using Tukey's post-hoc test (*p* < 0.05). Data are presented as mean values and standard deviations. The relationship between body weight and muscle parameters was assessed by correlation analysis.

## Results

### Food intake, body weight, drug intake and activity test

**Fig. 2:**
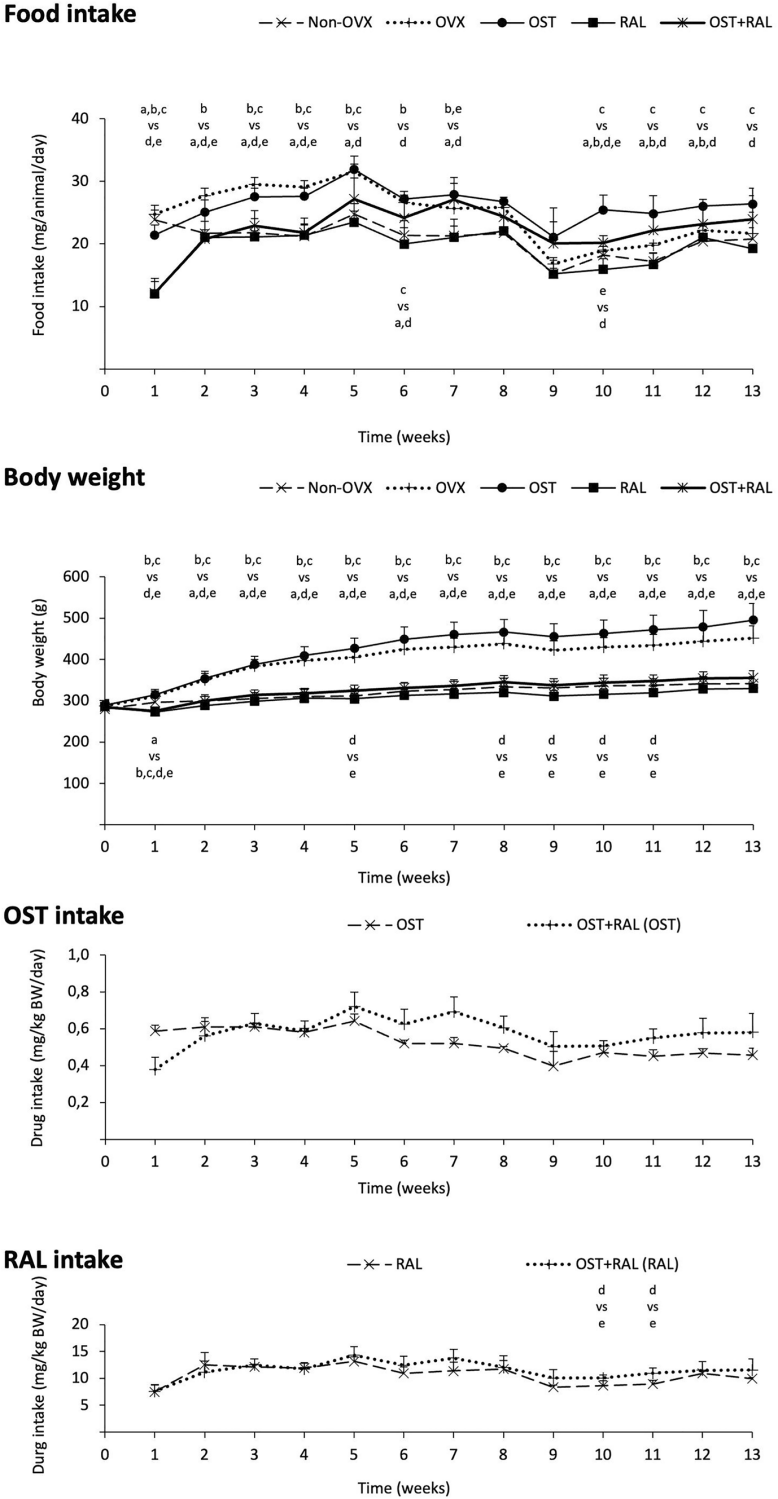
Food intake, body weight, and the intake of OST and RAL. The food intake, the body weight, and the drug intake during the experiment. Significant differences between the groups (**a** Non-OVX, **b** OVX; **c** OST; **d** RAL; **e** OST + RAL) are shown (*p* < 0.05, Tukey test)

Mean food intake was significantly different between the treatment groups (Table 1). The OVX and OST groups had significantly higher food intakes than the Non-OVX and RAL groups. Weekly food intake analysis showed higher food intake in the OVX and OST groups compared to all other groups for up to 8 weeks (Fig. 2). At week 10, higher food intake was particularly observed in the OST group compared to the other groups and in the OST + RAL group compared to the RAL group. Thereafter, it did not differ from that of the OST + RAL group, and at the end of the study, it differed only from that of the RAL group (Fig. 2).

**Table 1:** Food intake, weights, nucleus ratio, serum analysis, and activity test in Non-OVX or OVX rats treated either with ostarine (OST), raloxifen (RAL) or combination (OST + RAL)

Groups	Non-OVX		OVX		OST		RAL		OST + RAL		ANOVA *p*-value
Sample Size	13		15		15		15		15		
Parameters	Mean	SD	Mean	SD	Mean	SD	Mean	SD	Mean	SD	
Mean food intake (g/day/rat)	20.7^bc^	2.6	24.6^d^	4.5	26.1^d^	2.8	19.2	3.3	22.3	3.8	** < 0.001**
Weights											
Body weight (beginning of trial) [g]	271.5	12.8	276.7	11.9	273.0	11.3	271.6	11.6	269.7	8.0	0.518
Body weight (end of trial) [g]	341.5^bc^	17.4	451.5^cde^	30.5	495.1^de^	41.0	329.6	14.0	355.9	17.3	** < 0.001**
Uterus weight [g]	0.75^bd^	0.19	0.22^ce^	0.07	0.62^d^	0.09	0.25^e^	0.05	0.66	0.16	** < 0.001**
GM weight [g]	2.17^bc^	0.18	2.56^cde^	0.27	2.84^de^	0.18	2.07	0.21	2.23	0.18	** < 0.001**
SM weight [g]	0.18^c^	0.02	0.19^cd^	0.03	0.22^de^	0.03	0.16	0.02	0.17	0.02	** < 0.001**
Nucleus ratio											
GM	0.85^e^	0.24	0.95	0.20	1.07	0.37	0.94	0.21	1.13	0.34	**0.008**
LM	1.11	0.27	1.40	0.33	1.36	0.39	1.23	0.32	1.34	0.39	0.057
SM	1.21	0.43	1.06	0.28	1.04	0.25	1.19	0.37	0.94	0.25	0.068
Serum analysis											
Ca (mmol/l)	2.6^bcde^	0.1	2.5^e^	0.1	2.4	0.1	2.4	0.1	2.4	0.1	** < 0.001**
Mg (mmol/l)	0.9^bcde^	0.1	0.8	0.1	0.8	0.1	0.8	0.1	0.8	0.1	** < 0.001**
P (mmol/l)	1.6^cde^	0.2	1.7^ce^	0.2	2.0^e^	0.2	1.9^e^	0.2	2.3	0.2	** < 0.001**
CK (U/l)	799	264	825	460	540	248	1004	463	724	528	0.126
FSH (ng/ml)	16.41^de^	3.041	20.13	2.952	20.32	2.849	23.34	5.037	25.24	4.379	** < 0.001**
LH (pg/ml)	272.7^e^	44.49	358.3	169.7	369.1	70.17	335.2	110.6	457	104.1	**0.041**
Activity test											
Transition	54.1	8.2	62.6	9.4	54.5	9.2	60.9	10.6	50.6	8.2	0.093
Rearing	21.1	5.1	23.6	4.2	21.6	4.2	25.3	5.7	19.1	3.1	0.118
Grooming	3.4	1.5	4.5	1.6	3.1	1.4	2.6	1.7	2.8	1.6	0.140

*SD* Standard deviation, *STO + FTO* slow-twitch oxidative and fast-twitch oxidative fibers, *FTG* fast-twitch glycolytic fibers, *GM* gastrocnemius muscle, *LM* longissimus muscle, *SM* soleus muscle

^b^Different from OVX, ^c^From OST, ^d^From RAL,^e^From OST + RAL (Tukey test, *p* < 0.05)

The mean BW of the rats was highest in the OST group and higher in the OVX group than in the Non-OVX, RAL, and OST + RAL groups (Table 1). Similarly, BW was higher in the OVX and OST groups compared to all other groups throughout the experiment (Fig. 2). In addition, the BW of the RAL groups was significantly lower than that of the OST + RAL group at weeks 5, 8, 9, 10, and 11.

The mean OST intake was 0.52 ± 0.03 mg/kg BW for the OST group and 0.58 ± 0.07 mg/kg BW for the OST + RAL group (Fig. 2). The mean RAL intake was 10.62 ± 1.06 mg/kg BW for the OST group and 11.52 ± 1.41 mg/kg BW for the OST + RAL group. RAL intake was significantly higher in the OST + RAL group than in the RAL group during weeks 10 and 11 (Fig. 2).

None of the parameters recorded during the activity test (transitions, rearing and grooming) differed between groups (Table 1).

### Uterus and muscle weights

Uterine weight was significantly lower in the OVX and RAL groups compared with the Non-OVX, OST, and OST + RAL groups (Table 1).

Regarding muscle weight, GM and SM weights were significantly higher in the OST group compared to all other groups. The OVX group had a significantly higher SM weight compared to the RAL group and a higher GM weight compared to the Non-OVX, RAL, and OST + RAL groups (Table 1). Correlation analysis of muscle weight and BW showed a significant relationship between these variables in GM and SM (Table 3).

### Muscle structure analysis

In the GM, the diameter of STO + FTO fibers of the RAL group was significantly larger than that of the Non-OVX group. The diameter of FTG fibers was significantly larger in the OVX group and the OST group than in the OST + RAL group, and the OST group had a significantly larger diameter than the RAL group. The areas of all fibers did not differ between the treatment groups (Table 2). The size of FTG fibers correlated significantly with BW in the GM (Table 3).

**Table 2:** Muscle fiber analyses in Non-OVX or OVX rats treated either with ostarine (OST), raloxifen (RAL) or combination (OST + RAL)

Groups	Non-OVX		OVX		OST		RAL		OST + RAL		ANOVA *p*-value
Sample size	13		15		15		15		15		
Parameters	Mean	SD	Mean	SD	Mean	SD	Mean	SD	Mean	SD	
GM											
STO + FTO											
Area (µm^2^)	1597	452	1970	244	1938	303	1922	440	1849	315	0.159
Diameter (µm)	44^d^	6	50	3	49	4	51	7	48	4	**0.044**
FTG											
Area (µm^2^)	3964	900	4829	1193	4987	1059	3667	671	3576	902	0.108
Diameter (µm)	70	8	77^e^	9	78^de^	8	68	6	67	8	**0.004**
LM											
STO + FTO											
Area (µm^2^)	1669	294	1900^de^	435	1704	253	1479	258	1498	309	**0.011**
Diameter (µm)	46	4	50^de^	7	46	4	43	4	43	4	**0.008**
FTG											
Area (µm^2^)	4738	810	5920	1323	6187	1400	5218	1202	5413	1297	0.055
Diameter (µm)	77	7	88	11	88	11	80	9	82	10	0.060
SM											
STO											
Area (µm^2^)	4079	593	4214^d^	470	4097	322	3439	414	3716	924	**0.026**
Diameter (µm)	72	5	73	4	72	3	66	4	68	8	0.224
Percentage of fibers in LM (%)											
STO + FTO	50	7	52	8	49	7	54	6	48	4	0.181
FTG	50	7	48	8	51	7	46	6	52	4	0.181

*SD* Standard deviation, *STO + FTO* slow-twitch oxidative and fast-twitch oxidative fibers, *FTG* fast-twitch glycolytic fibers, *GM* gastrocnemius muscle, *LM* longissimus muscle, *SM* soleus muscle

^d^Different from RAL,^e^From OST + RAL (Tukey test, *p* < 0.05)

**Table 3:** Correlations between body weight (BW) and muscle parameters

Correlations between BW and …		Pearson *r*	*R*^2^	*p*
GM	Weight	0.83	0.68	< 0.001
	STO + FTO diameter	0.15	0.021	0.330
	STO + FTO area	0.22	0.047	0.145
	FTG diameter	0.49	0.24	** < 0.001**
	FTG area	0.49	0.24	** < 0.001**
	Capillary ratio	0.33	0.11	**0.031**
	Nucleus ratio	0.12	0.014	0.414
LM	STO + FTO diameter	0.34	0.11	**0.020**
	STO + FTO area	0.29	0.084	**0.048**
	FTG diameter	0.36	0.13	**0.011**
	FTG area	0.33	0.11	**0.021**
	Capillary ratio	0.50	0.25	** < 0.001**
	Nucleus ratio	0.30	0.089	**0.046**
SM	Weight	0.66	0.43	** < 0.001**
	STO + FTO diameter	0.28	0.077	0.060
	STO + FTO area	0.26	0.067	0.077
	Capillary ratio	0.27	0.076	0.075
	Nucleus ratio	-0.20	0.042	0.184

*STO + FTO* slow-twitch oxidative and fast-twitch oxidative fibers, *FTG* fast-twitch glycolytic fibers, *GM* gastrocnemius muscle, *LM* longissimus muscle, *SM* soleus muscle, *R*^2^: Coefficient of determination, *p*: two-tailed *P*-value

In the LM, the areas and diameters of STO + FTO fibers were significantly larger in the OVX group than in the RAL and OST + RAL groups, whereas those of FTG fibers did not differ between groups (Table 2). All fiber types examined correlated positively with the BW of the rats (Table 3). The ratio of STO + FTO fibers to FTG did not differ significantly between the groups in the LM (Table 2).

In the SM, the area of STO fibers was significantly larger in the OVX group than in the RAL group (Table 2). There was no correlation between fiber size and BW of the rats (Table 3).

**Fig. 3:**
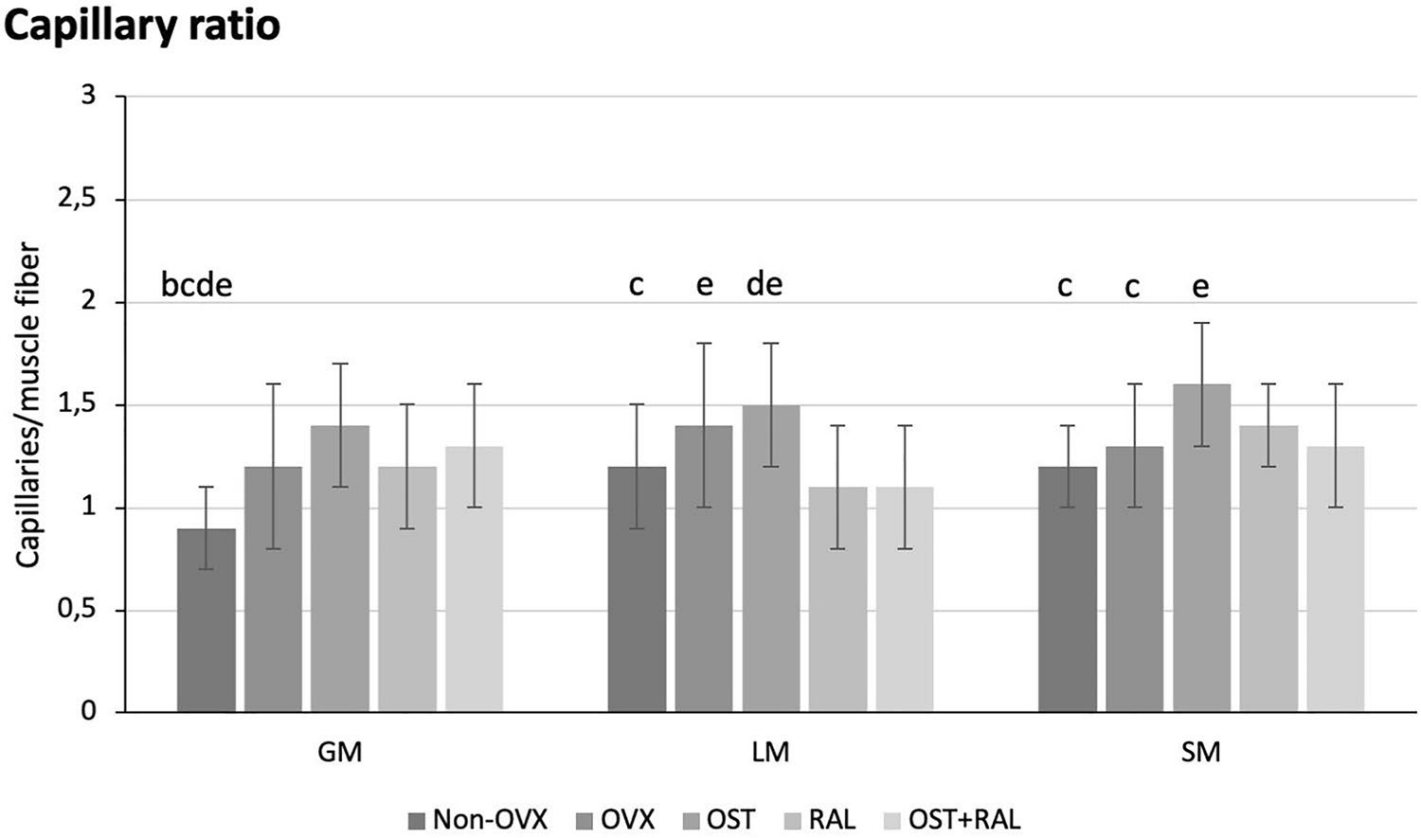
Capillary ratio. The capillary ratio in the GM, LM and SM is shown. **b** Capillary ratio is significantly different from the OVX group, **c** from the OST group, **d** from the OST group, and **e** from the OST + RAL group (*p* < 0.05, Tukey test)

Regarding the capillary ratio, the Non-OVX group had a significantly lower ratio than all other groups in the GM (Fig. 3). In the LM, the OVX group and the OST group had a significantly higher ratio compared to the OST + RAL group. In addition, the OST group had a significantly higher ratio compared to the Non-OVX and RAL groups (Fig. 3). In the SM, a significantly higher ratio was observed in the OST group compared to the Non-OVX, OVX, and OST + RAL groups (Fig. 3). In the GM and LM, the capillary ratio was significantly correlated with BW (Table 3).

The analysis of the nucleus ratio (nuclei per muscle fiber) was significantly higher in the OST + RAL group than in the Non-OVX group in the GM. There were no significant differenes in the LM or SM (Table 1).

Correlations between nucleus ratio and BW were not significant for GM and SM, but reached a significant level for LM (Table 3).

### Serum analysis

Ca and Mg levels were significantly higher in the Non-OVX group compared to all other groups. In addition, the OVX group had a significantly higher Ca level than the OST + RAL group (Table 1). The P level of the OST + RAL group was the highest among the other treatment groups. It was significantly lower in the Non-OVX group than in the OST and RAL groups, and lower in the OVX group than in the OST group (Table 1). There were no significant differences in CK activity (Table 1). In hormonal analysis, significantly higher FSH levels were observed in the RAL and OST + RAL groups compared to the Non-OVX group. LH levels were also significantly higher in the OST + RAL group than in the Non-OVX group (Table 1).

### Gene expression analysis

**Fig. 4:**
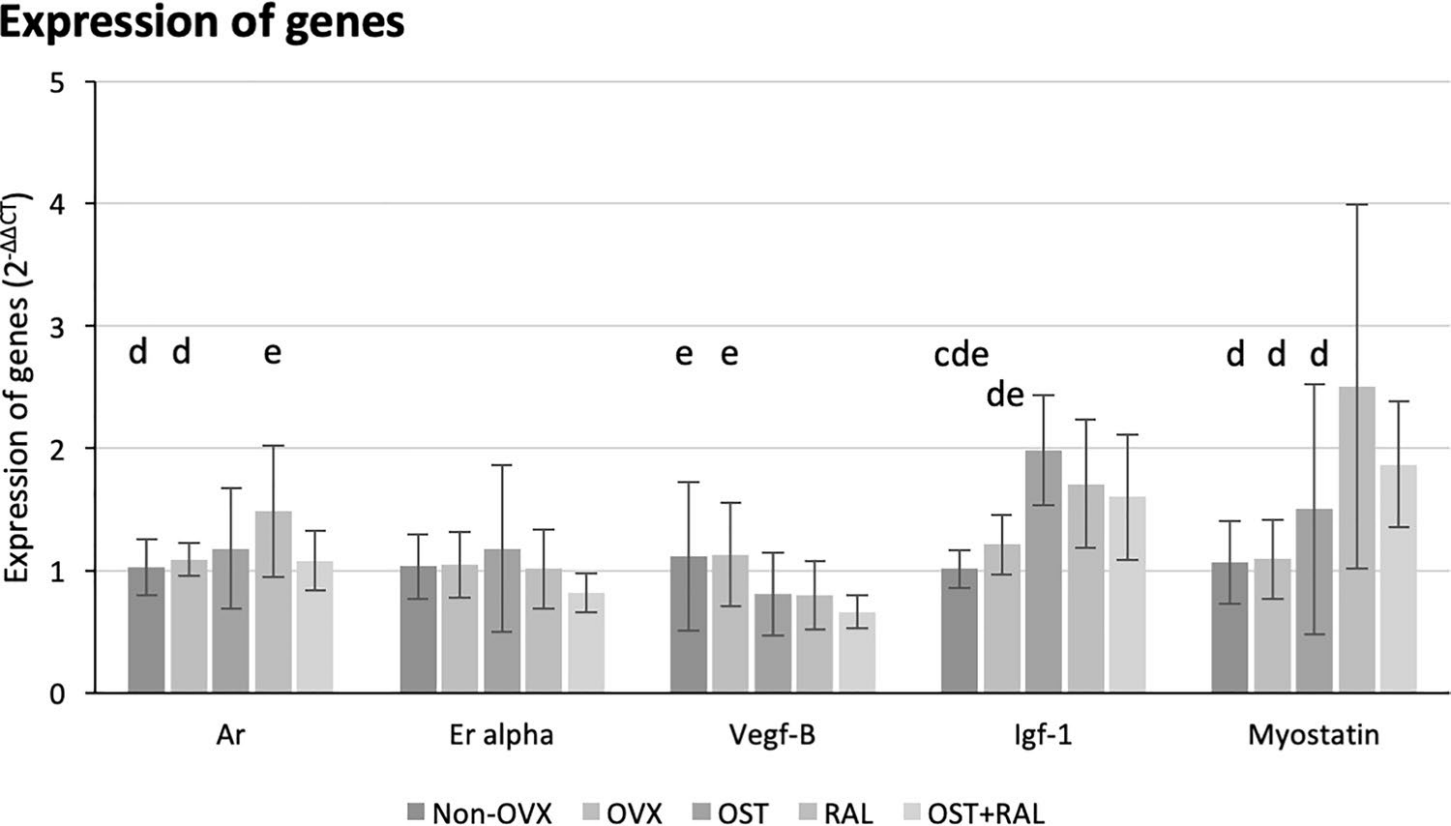
Expression of genes. Gene expression in the GM is shown. **c** Gene expression is significantly different from the OST group, **d** from the OST group, and **e** from the OST + RAL group (*p* < 0.05, Tukey test)

Ar gene expression was significantly higher in the RAL group compared to the Non-OVX, OVX and OST + RAL groups (Fig. 4). No significant differences were found in the Er alpha expression. Vegf-B gene expression was significantly higher in the Non-OVX, and OVX groups than in the OST + RAL group. Igf-1 expression was higher in the OST, RAL, and OST + RAL groups than in the Non-OVX group. Igf-1 gene was expressed expression was significantly higher in the OST and RAL groups than in the OVX group. Myostatin expression was significantly higher in the RAL group than in the Non-OVX, OVX, and OST groups (Fig. 4).

## Discussion

In this study, we analyzed the effects of the SARM ostarine and the SERM raloxifen on muscle structure and metabolism in ovariectomized rats as a model of postmenopausal musculoskeletal system deterioration. We found that OST treatment exerted beneficial effects on muscle tissue, whereas RAL or combined OST + RAL treatments had less effect on muscle.

After OVX, rats showed increased BW as a common response to estrogen deprivation, which is consistent with previous studies [11, 32–35] and could be explained by an increased food intake and other metabolic changes in rats observed after OVX [36]. While OST + RAL treatment did not alter BW in the OVX rats, RAL administration resulted in a decrease in food intake and BW, possibly similar to the mechanisms of estrogens in blunting the increase in BW [36]. OST administration did not change BW throughout the experiment; however, during the last four weeks of the experiment, OST rats showed increased food intake. It is possible that changes in BW and food intake are time dependent. This would be consistent with the results of Kearbey, Gao [37], who found increased BW in OVX rats after 120 days of SARM S-4 administration, while body fat was reduced and lean mass was increased. Overall, the rats in the present study had a higher food intake than in our previous study [11], which resulted in a higher uptake of the test compounds. However, the doses were comparable to the other rodent studies [38, 39].

The activity of the rats was not affected by any of the treatments. Previous studies have reported a decrease in physical activity in OVX rats that promotes weight gain [40, 41], whereas estrogen replacement therapy has been associated with a return to normal physical activity and body weight [40]. The limitations of the physical activity assessment test used in this study are its short duration and application time at the end of the experiment. We did not perform more comprehensive tests because analysis of physical activity and behavior were not the primary objectives of the study.

OST treatment alone and in combination resulted in increased uterine weight, which has been previously observed and considered as a negative side effect [11, 34, 42]. One reason for this uterotrophic effect may be that different scaffolds interact with the N-/C-terminal domains of the androgen receptor, leading to reduced tissue selectivity [43]. In addition, OST was shown to increase the number of Ki67-positive cells in the mouse uterine stroma and epithelial cell proliferation [39]. We did not observe any effects of RAL on uterine weight, which could be seen in line with its more estrogen-antagonistic potential on the uterus [44].

OST treatment resulted in muscle weight gain in the GM and MS, highlighting the anabolic effect of SARM. A metabolic explanation for the ostarine-induced muscle weight gain in rats could be the stimulation of muscle cell differentiation by increasing the expression of myogenin, myoblast determination protein 1, and myosin heavy chain [45]. Dalton, Barnette [14] showed an increase in lean body mass and a decrease in total fat mass in postmenopausal women after OST treatment, supporting our findings. In contrast to OST, RAL treatment alone or in combination with OST maintained muscle weight at the level of intact Non-OVX rats, thereby reducing the effect of OVX. The changes in muscle weight could be due to the differences in BW of these groups, since the effect was not seen in data expressed relative to BW (data not shown) and a high positive correlation was found between muscle and body weight. Indeed, SERMs have been shown to improve muscle function and structure in mice with muscular dystrophy, possibly due to reduced fibrosis, oxidative stress, and mitochondria-mediated cell death [17, 46]. Shen [47] observed reduced BW in OVX rats treated with RAL and postulated regulation of the Wnt signaling pathway and a subsequent inhibition of adipogenesis.

OST administration did not affect muscle fiber size. The RAL group or RAL + OST group partially showed decreased muscle fiber sizes (e.g. STO + FTO in LM). A similar effect of RAL treatment alone or in combination was observed in previous experiments in male rats [12]. A possible explanation could be a decrease in BW under RAL treatment, as muscle weight and muscle fiber size correlate with BW in rats in the present and previous study [48].

Consistent with previous findings in OVX rats [11], OST administration in the present study showed beneficial effects on capillary ratio in the LM and SM. Thus, we report an increased capillary ratio after OST administration in female OVX rats, which may indicate that female muscles are more sensitive to OST than male muscles studied by Roch, Wolgast [12]. Better vascularization due to increased capillary ratio influences the recovery of muscle contractility and may subsequently improve muscle function [49]. In contrast to OST, RAL treatment and the combined treatment of RAL and OST resulted in a decreased capillary ratio in the LM. The expression of Vegf-B, which influences muscle vascularization [50] in our study, was least expressed in the combined treatment. Capillary ratio was correlated with BW and correspondingly with muscle weight, which may explain the lower blood supply in these treatment groups. In other studies, when RAL or estrogen was used as a therapeutic treatment 8 weeks after OVX, no changes in capillary ratio in skeletal muscles were reported [51, 52].

In regard to the nucleus ratio, it was increased only in the GM by the combined treatment compared to the Non-OVX group. An increase in the number of satellite cells and myonuclei is associated with testosterone-induced muscle fiber hypertrophy [53]. In our study, neither OST nor RAL was shown to affect the amount of myonuclei, although in general all OVX groups had a slightly non-significant higher ratio of myonuclei in muscle than Non-OVX rats.

Gene expression analysis showed increased Ar gene expression in the GM in the RAL group compared to the OVX controls. In contrast, decreased Ar expression has been reported in orchiectomized males after RAL administration [12]. Sex differences may contribute to the differential expression of Ar in muscle. Igf-1 gene expression was increased in the RAL and RAL + OST groups. Igf-1 activates the calcium-dependent calcineurin signaling pathway in skeletal muscle, thereby promoting muscle growth [54]. Thus, a similar effect of the combination treatment on Igf-1 expression as previously reported [12] was confirmed. Tsai, McCormick [55] observed that reduced estrogen levels after OVX in rats resulted in higher Igf-1 expression, possibly indicating its role in mediating the effects of estrogen deprivation. Furthermore, they showed that Igf-1 protein level decreased and Myostatin protein level increased after estrogen replacement [55]. In our study, Myostatin expression was also increased after treatment of OVX rats with RAL, whereas OST and OST + RAL treatments did not change its expression. In male orchiectomized rats, OST administration resulted in a decreased Myostatin expression, whereas RAL treatment did not affect Myostatin expression [12]. Myostatin controls muscle growth by inhibiting muscle differentiation and growth [56, 57], which may explain the inhibition of muscle weight gain after ovariectomy in the RAL and OST + RAL groups.

Serum analysis showed that Ca and Mg levels were lower in all OVX groups than in the healthy Non-OVX group, with the combined treatment reducing Ca levels to a greater extent. In contrast, P levels were generally higher after OVX and increased significantly after OST and OST + RAL treatments. One explanation could be an effect of ovariectomy on the thyroid and the hypothalamic-pituitary-thyroid axis [58]. While the changes in serum after OVX have been reported previously [34, 59], the combination treatment failed to restore P and Ca levels as it was observed in male orchiectomized rats [12]. Shahida [60] found low Ca levels in osteoporotic patients, which may be due to the decrease in estrogen levels during menopause [61]. Electrolyte imbalance should be avoided to prevent serious complications [62] and this side effect of combined treatment should be considered. None of the treatments showed an effect on CK levels, indicating a lack of muscle damage [63].

Serum FSH and LH levels were generally higher in all OVX rats compared to the Non-OVX rats, reaching the highest levels after the combination treatment. Both hormones, FSH and LH are elevated after the menopause in women and OVX rats due to the decreased estrogen and inhibin levels [64]. Estrogen and RAL decrease LH levels by suppressing gonadotropin-releasing hormone (GnRH) release [65], whereas FSH levels may not be decreased [66]. Androgenic steroid hormones and SARMs also have the potential to suppress of LH and FSH levels [67] and it is unclear why the combination of RAL and OST caused an increase in the levels of these hormones.

The study has several limitations. We examined metabolism, structure and gene expression and did not include a functional examination of the muscles. For clinical application, the effects of substances on muscle function should be addressed. In addition, the effects of anabolic substances on muscle metabolism and size may be influenced by concomitant exercise [68, 69], which was not assessed in the present study. Furthermore, the Non-OVX rats did not experience surgical stress and postoperative pain, and therefore the sham-operated group would have been a more appropriate control group for this study.

Summarizing, OST administration resulted in favorable effects on muscle weight and capillary ratio, emphasizing the anabolic effect of SARMs, while RAL or combination therapy failed to do so. Neither treatment showed anabolic effects on muscle fiber size. The combination treatment increased the nucleus ratio in the GM compared to the Non-OVX group. OST administration did not change BW, whereas RAL administration reduced food intake and BW, consistent with the literature. In contrast to RAL, OST administration and the combination treatment increased uterine weight and had altered serum electrolyte concentration, suggesting possible side effects due to the limited tissue selectivity. Gene expression analysis revealed beneficial effects on muscle growth as indicated by increased Igf-1 expression after OST and RAL administration. However, RAL treatment also increased Myostatin expression, which likely slowed muscle growth and prevented the increase in muscle weight observed after OVX.

In conclusion, the effect of OST on muscle was favorable and superior to the effect of RAL alone or combined treatment in estrogen-deficient rats. However, side effects of OST on uterus and serum electrolytes should be considered before using it for therapeutic purposes. RAL and RAL + OST had less effect on muscle and showed some endocrinological side effects on pituitary–gonadal axis.
